# Proteomic analysis of meropenem-induced outer membrane vesicles released by carbapenem-resistant *Klebsiella pneumoniae*

**DOI:** 10.1128/spectrum.02917-23

**Published:** 2024-01-18

**Authors:** Fangfang Fan, Guangzhang Chen, Siqian Deng, Li Wei

**Affiliations:** 1 Anhui Key Laboratory of Infection and Immunity, Bengbu Medical College, Bengbu, China; University of Florida, Gainesville, Florida, USA

**Keywords:** CRKP, antibiotic resistance, outer membrane vesicle, proteomics, protein-protein interactions

## Abstract

Carbapenem-resistant *Klebsiella pneumoniae* (CRKP) is an important multidrug resistance (MDR) pathogen that threatens human health and is the main source of hospital-acquired infection. Outer membrane vesicles (OMVs) are extracellular vesicles derived from Gram-negative bacteria and contain materials involved in bacterial survival and pathogenesis. They also contribute to cellular communication to nearby or distant recipient cells and influence their functions and phenotypes. In this study, we sought to understand the mechanism of bacterial response to meropenem pressure and explore the relationship between pathogenic proteins and the high pathogenicity of bacteria. We performed whole-genome PacBio sequencing on a clinical CRKP strain, and its OMVs were characterized using nanoparticle tracking analysis, transmission electron microscopy, and proteomic analysis. Thousands of vesicle proteins have been identified in mass spectrometry-based high-throughput proteomics analyses of *K. pneumoniae* OMVs. Protein functionality analysis showed that the OMVs were predominantly involved in metabolic, intracellular compartments, nucleic acid binding, survival, defense, and antibiotic resistance, such as Chromosome partition protein MukB, 3-methyl-2-oxobutanoate hydroxymethyltransferase, methionine—tRNA ligase, Heat shock protein 60 family chaperone GroEL, and Gamma-glutamyl phosphate reductase. Additionally, a protein-protein interaction network demonstrated that OMVs from meropenem-treated *K. pneumoniae* showed the highest connectivity in DNA polymerase I, phenylalanine–tRNA ligase beta subunit, DNA-directed RNA polymerase subunit beta, methionine–tRNA ligase, DNA-directed RNA polymerase subunit beta, and DNA-directed RNA polymerase subunit alpha. The OMVs proteome expression profile indicates increased secretion of stress proteins released from meropenem-treated *K. pneumoniae*, which provides clues for revealing the biogenesis and pathophysiological functions of Gram-negative bacteria OMVs. The significant differentially expressed proteins identified in this study are of great significance for exploring effective control strategies for CRKP infection.

**IMPORTANCE:**

Meropenem is one of the main antibiotics used in the clinical treatment of carbapenem-resistant *Klebsiella pneumoniae* (CRKP). This study demonstrated that some important metabolic changes occurred in meropenem-induced CRKP-outer membrane vesicles (OMVs), The OMVs proteome expression profile indicates increased secretion of stress proteins released from meropenem-induced *Klebsiella pneumoniae*. Furthermore, this is the ﬁrst study to discuss the protein-protein interaction network of the OMVs released by CRKP, especially under antibiotic stress.

## INTRODUCTION

Carbapenem-resistant *Klebsiella pneumoniae* (CRKP) causes significant worldwide morbidity and mortality and is of paramount concern in healthcare-associated infection settings (1). Due to limited treatment options, when the patient is infected with ESBLs-producing bacteria, if the use of third-generation cephalosporin/enzyme inhibitors is ineffective, only carbapenem drugs (meropenem, imipenem, and ertapenem) can be used for treatment. Even the use of such a limited range of antibiotics is more likely to lead to carbapenem-resistant bacteria, it leads to a high incidence rate and mortality. As a carbapenem-sparing antibiotic, meropenem is widely used for the treatment of numerous infections. The principal parameter correlated to the success of therapy is the percentage of time that the levels are maintained above the minimum inhibitory concentration (MIC). Inadequate levels of meropenem would contribute to therapeutic failure and enhance the risk of microbial resistance development. So, the reasonable therapies require optimized strategies involving dose regimens and drug pharmacodynamics (2). Since the identification of *K. pneumoniae* isolates harboring carbapenemase in 1996, global dissemination of CRKP has occurred (3). Infections caused by CRKP are difficult to treat since these isolates often harbor resistance to multiple antibiotics, severely limiting therapeutic options (4). CRKP strains account for the majority of clinical carbapenem-resistant Enterobacteriaceae (CRE) infections (5, 6). Due to the inappropriate use and abuse of antibiotics in recent years, drug resistance, especially the multidrug resistance (MDR) of bacteria, has become one of the main threats to global public health (7–10). Antimicrobial use and overuse are important drivers of the gradually increasing drug resistance. In addition, some factors that promote the dissemination of resistant bacteria and their genes locally and globally are also drivers (11).

Outer membrane vesicles (OMVs) are predominantly spherical bilayered nanostructures filled with periplasmic content and are commonly released from Gram-negative bacteria. OMVs biogenesis facilitates bacteria to interact with the external environment and mediate multiple functions, including promoting pathogenesis, allowing bacterial survival under stress conditions, and regulating interactions within communities (12). Various stress conditions, especially in punitive environments, can modify the vesiculation of OMVs. Furthermore, OMVs serve as an envelope stress response (13).

Moreover, the biogenesis mechanism and content of OMVs under stress conditions seem to be different from those produced under normal conditions. Bacterium-secreted OMVs enable bacterial communication and regulate host immune effects (14). The presence of OXA-58 in carbapenem-resistant *Acinetobacter baumannii* OMVs was confirmed using carbapenem inactivation assay and proteomic analysis techniques. A recent study demonstrated that imipenem treatment can promote OMVs formation and cause cell lysis, increasing the release of extracellular OXA-58 (15). Infections caused by CRKP are associated with more disease complications and higher mortality rates (16). OMVs contain various virulence factors, including toxins, enzymes, and adhesins, which can affect the infection process and host immune response (17). However, the changes in OMVs secretion, resistance, pathogenicity, and other related proteins of *K. pneumoniae* under antibiotic stress need to be evaluated.

Bioinformatics analysis of mass spectrometry-based proteomics data has provided an opportunity to combat antibiotic resistance and reveal unknown functional relationships (18). This study describes the morphology and content of OMVs released from CRKP after meropenem treatment. Understanding the physical characteristics, protein content, and possible metabolic function of OMVs secreted by these bacteria will help to understand the mechanism of bacteria responding to environmental pressure.

## RESULTS

### Antimicrobial susceptibility is determined by the minimum inhibitory concentration

*K. pneumoniae* is resistant to carbapenems was isolated from sputum cultures of a 52-year-old female neurosurgical patient with intraventricular hemorrhage. The minimum inhibitory concentration(MIC) of the strain using the broth microdilution method for 13 antibiotics revealed resistance (R), susceptibility (S), and an intermediate (I) pattern. The instrument used for strain drug sensitivity testing is VITEK. The strain was resistant to amoxicillin/clavulanic acid (AMC; MIC ≥32 µg/mL), piperacillin/tazobactam (TZP; MIC ≥128 µg/mL), cefuroxime(injection) (CXM; MIC ≥ 64 µg/mL), cefuroxime(oral) (CXM; MIC ≥64 µg/mL), ceftazidime (CAZ; MIC ≥64 µg/mL), ceftriaxone (CRO; MIC ≥64 µg/mL), cefepime (FEP; MIC ≥32 µg/mL), cefoxitin (FOX; MIC ≥64 µg/mL), imipenem (IMP; MIC ≥16 µg/mL), ertapenem (ETP; MIC ≥8 µg/mL), meropenem (MEM; MIC ≥4 µg/mL), amikacin (AMK; MIC ≥64 µg/mL), and compound sulfamethoxazole (SXT; MIC ≥16/304 µg/mL) (Table 1).

**TABLE 1:** Antimicrobial susceptibility proﬁles of the *K. pneumoniae* strain

Antibiotics	Results (μg/mL）	Susceptibility	Breakpoint
AMC	≥32	R	8–32
TZP	≥128	R	16–128
CXM (injection)	≥64	R	4–32
CXM (oral)	≥64	R	8–32
CAZ	≥64	R	4–16
CRO	≥64	R	1–4
FEP	≥32	R	2–16
FOX	≥64	R	8–32
IPM	≥16	R	1–4
ETP	≥8	R	0.5–2
MEM	≥4	R	1–4
AMK	≥64	R	16–64
SXT	≥16/304	R	2–4

### Genotyping of isolates

We conducted whole-genome PacBio sequencing analysis on the isolated strains and analyzed the genotype and plasmid of the isolates. The CRKP isolate belongs to ST11 (sequence types) that produces KPC-2. According to the database records, ST11 strains are commonly isolated in China. The clinical CRKP strain expresses the blaKPC-2 gene carried by the plasmid (plasmid IncFII) (Table 2).

**TABLE 2:** Analysis of multilocus sequence typing results (MLST）

Locus	Allele	Length	Contig	Start position	End position
gapA	3	450	Contig00001	3154972	3155421
infB	3	318	Contig00001	568950	569267
mdh	1	477	Contig00001	511385	511861
pgi	1	432	Contig00001	5075711	5076142
phoE	1	420	Contig00001	4279568	4279987
rpoB	1	501	Contig00001	5135101	5135601
tonB	4	414	Contig00001	2151455	2151868

### CRKP secretes OMVs during *in vitro* growth

**Fig 1:**
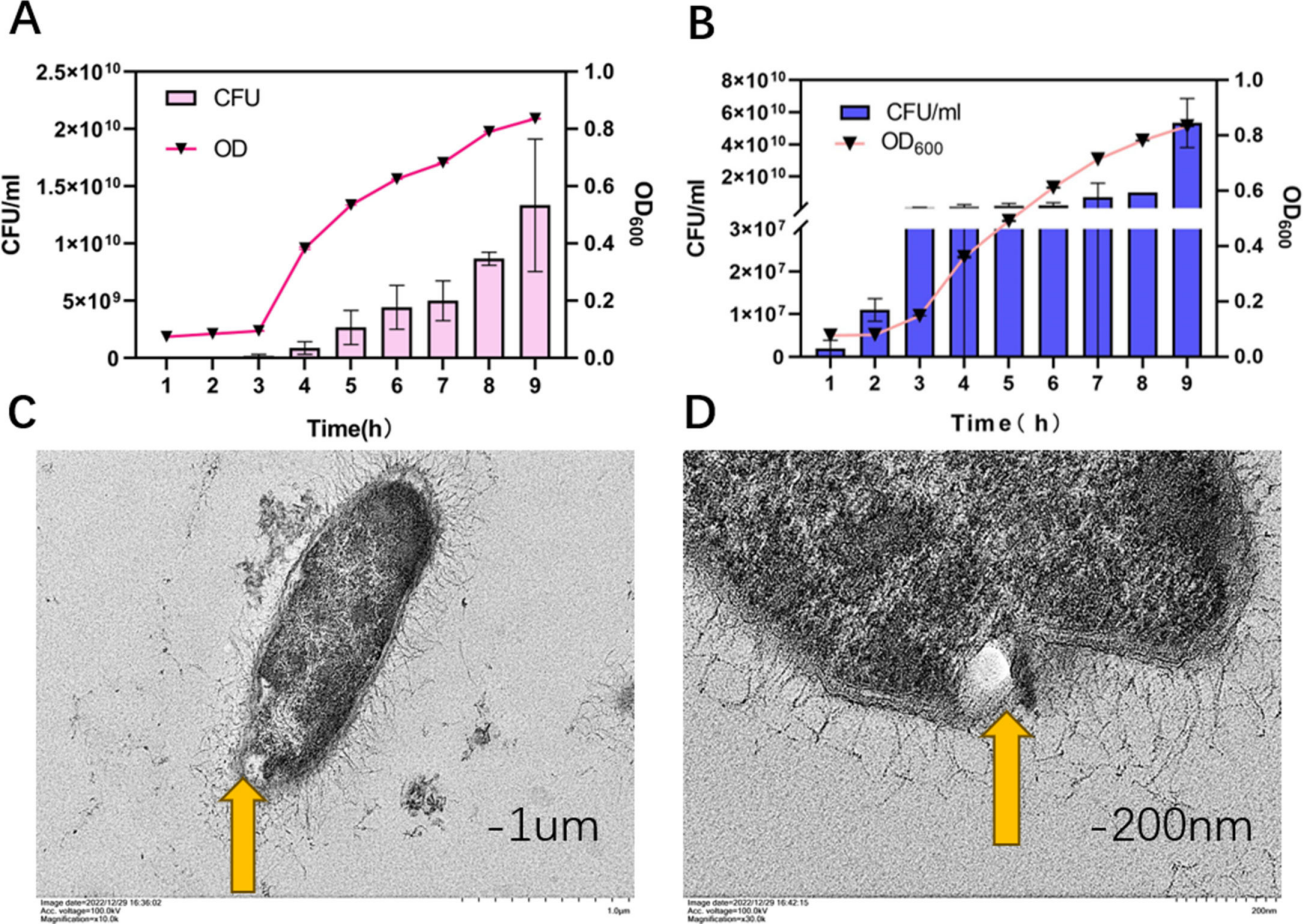
Growth of CRKP and CRKP induced by meropenem, and TEM observation of CRKP-OMV formation. MIC was used for meropenem (4 μg/mL). Data were representative of the results for three independent OMV preparations. (A) Growth of CRKP in LB medium. Time points were taken every 1 h for 9 h. Error bars were represented as means ± SD. Data points as shown were the averages from three separate experiments. (B) Growth of CRKP induced by meropenem in LB medium. Time points were taken every 1 h for 9 h. Error bars were represented as means ± SD. Data points as shown were the averages from three separate experiments. TEM showed individual OMV vesiculation from mid-log-phase CRKP cultures (orange arrows). Scale bar: (C) 1.0 μm and (D) 200 nm.

OMVs production by CRKP and meropenem-induced CRKP were measured during log-phase growth to reduce contamination of membrane proteins and cytoplasmic components caused by cell lysis debris during the stage of stabilization (Fig. 1A and B). Thus, after 8 h of growth, OMVs were harvested, and purified by density gradient ultracentrifugation and then characterized and visualized by transmission electron microscopy (TEM). OMVs blebbing from the outer membrane was observed (Fig. 1C and D), These results revealed that CRKP could secrete OMVs during *in vitro* growth.

### Purification and physical characterization of OMVs

**Fig 2:**
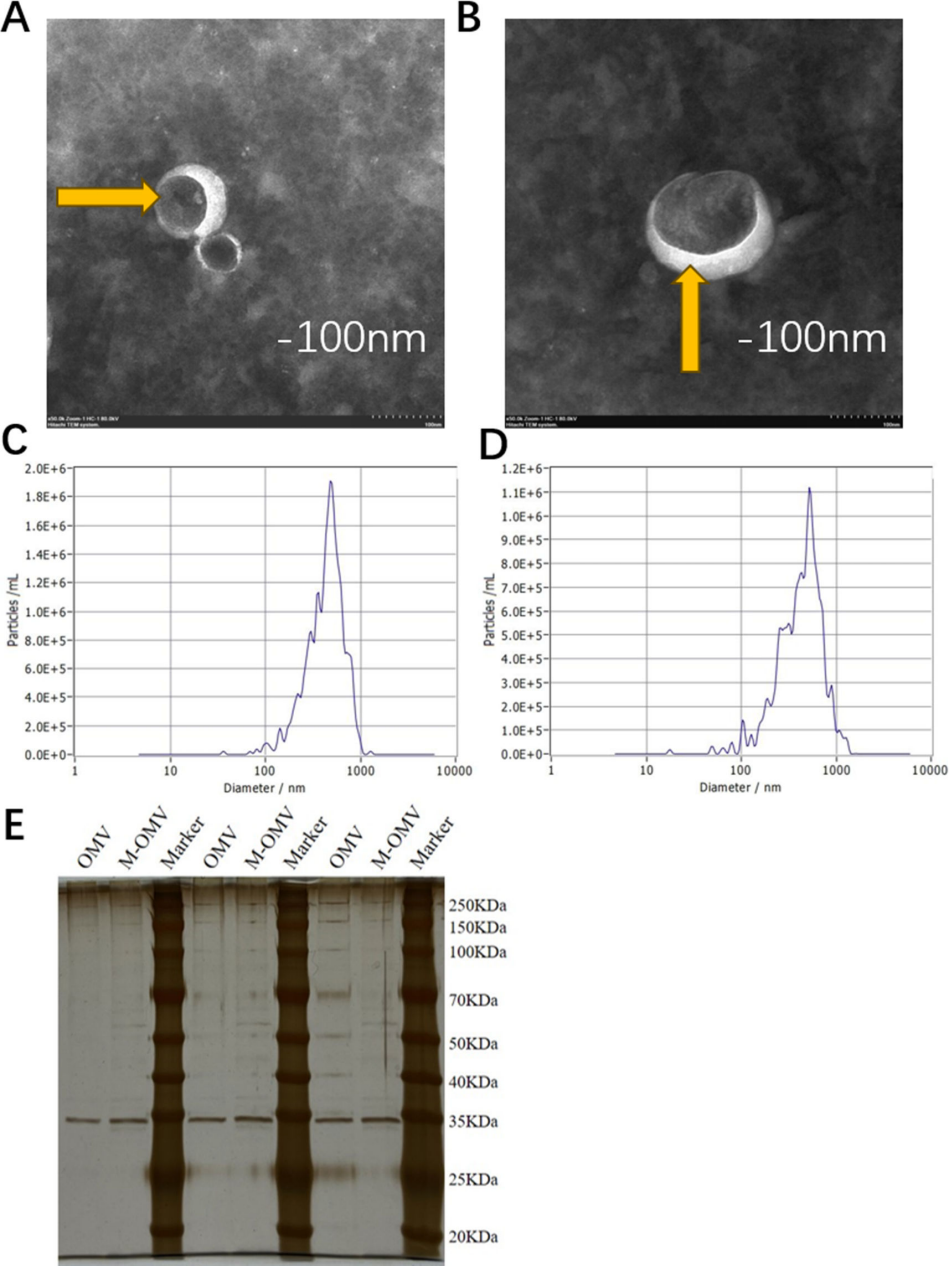
OMV characteristics, protein concentrations determination, and SDS-PAGE. TEM images of OMVs (orange arrows) and NTA of OMVs. (A and B) TEM images of OMV (A) and M-OMV (B) of CRKP vesicle samples. Scale bar: 100 nm. (C) NTA of OMV and (D) NTA of M-OMV. (E) Silver-stained SDS-PAGE of the OMVs. About 1 μg of total protein loaded in each lane.

To determine the physical characteristics of CRKP OMVs under antibiotic pressure, we collected OMVs after treatment with and without meropenem at MIC at 600 nm (OD_600_) = ~0.8 for 8 h and measured particle concentration, size distribution, and composition. The control group was OMV without antibiotic induction, represented by OMV, and the treated group was OMV with meropenem induction, represented by M-OMV. TEM showed that the purified OMVs had a unique spherical structures with a complete bilayer membrane and characteristic electron dense contents (Fig. 2A and B). Nanoparticle tracking analysis (NTA) results showed that the particle concentration of OMV was 8.6 × 9^10^ particles/mL, with a median size of 447.6 nm, while the particle concentration of M-OMV was 5.7 × 9^10^ particles/mL, with a median size of 442.1 nm (Fig. 2C and D). The OMVs were well purified and no bacterial growth was observed on the LB agar medium.

The mean protein concentration of the OMV and M-OMV were 924.8 µg/mL and 783.3 µg/mL, respectively. SDS-PAGE analysis showed that both OMV and M-OMV preparations showed numerous protein bands with a heterogeneous pattern (Fig. 2E).

### Proteomic analysis of meropenem-induced OMVs released by CRKP

**Fig 3:**
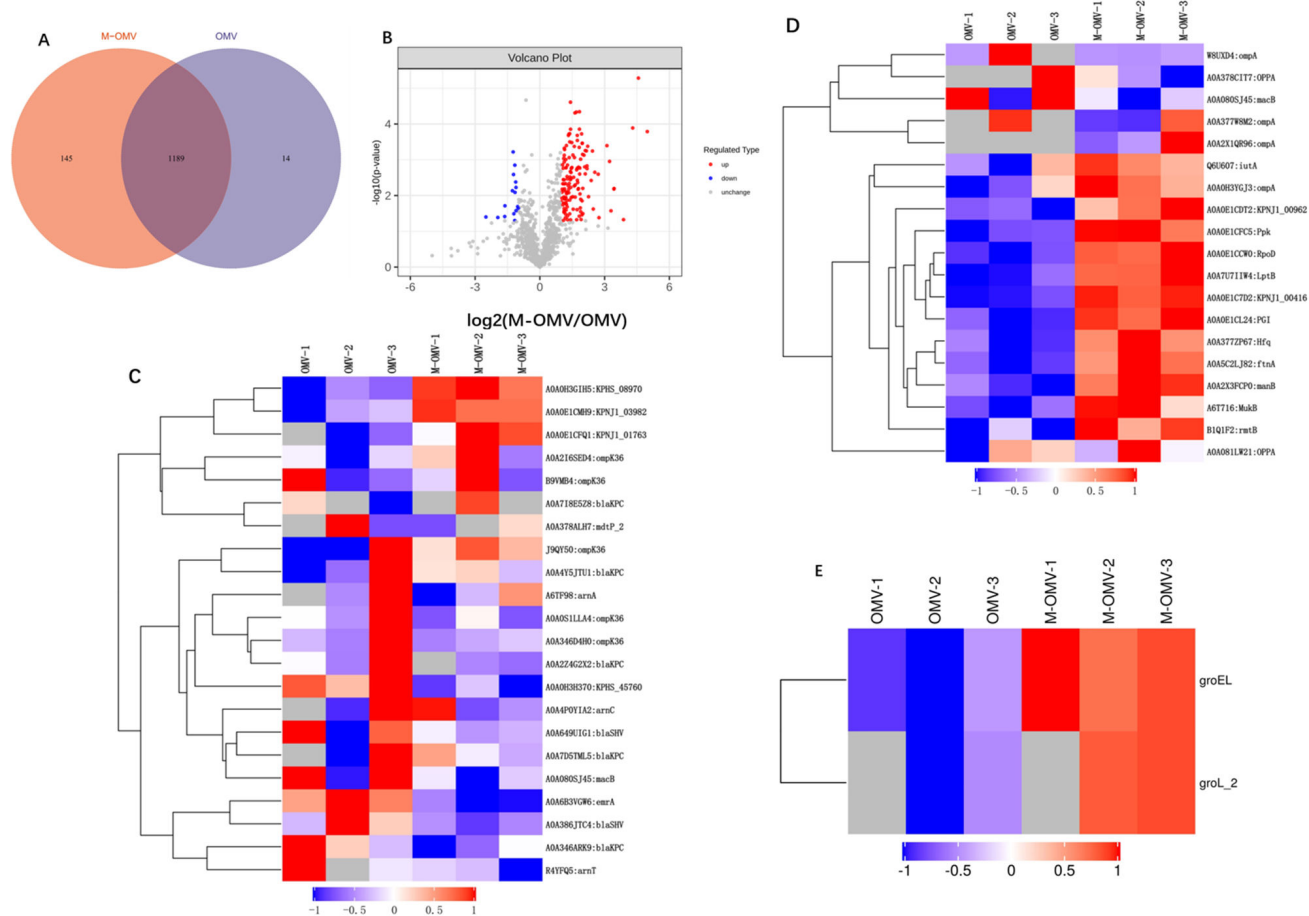
Comparison of OMVs proteins derived from meropenem-treated and control strains of CRKP. (A) Venn diagram of differentially expressed proteins according to proteomic analysis. A total of 1,203 proteins for OMV and 1,334 proteins for M-OMV were identiﬁed, of which 145 proteins were uniquely expressed in the meropenem-treatment model. Fourteen proteins were unique to the control, and 1,189 were differentially expressed. (B) Volcano map of significantly differentially expressed proteins. The abscissa was the multiple of the difference (logarithmic transformation based on the base of 2), and the ordinate was the significant *P* value of the difference (logarithmic transformation based on the base of 10). The red dot in the figure was the upregulated significant differentially expressed protein (FC > 2 and *P* < 0.05), the blue dot was the downregulated significant differentially expressed protein (FC < 0.50 and *P* < 0.05), and the gray dot indicates that no protein has been identified. (C) Clustering analysis of resistance proteins. (D) Clustering analysis of virulence proteins. (E) Clustering analysis of stress protein.

The mass spectrometry experimental analysis process mainly includes protein extraction, peptide enzymolysis, chromatographic classification (optional), liquid chromatography-tandem mass spectrometry (LC-MS/MS) data collection, database retrieval, and other steps. Proteins from OMVs were in-gel digested with trypsin and extracted from the gel for proteomic analysis. The complete proteome identiﬁed totals of 1,203 and 1,334 proteins from the control and M-OMV samples, respectively. Among these proteins, 1,189 were identiﬁed in both control and treated OMVs samples, while 145 and 14 unique proteins were identiﬁed in the treated and untreated OMVs, respectively (Fig. 3A). In the analysis of significance difference, the number of upregulated proteins was 139, and the number of downregulated proteins was 16. A volcano plot indicated the significant differences in proteins between the control and treated groups (Fig. 3B). For clustering analysis of interacting proteins, Cluster analysis of related resistance proteins between the two groups showed that two resistance proteins, Outer membrane channel-Porin superfamily protein and Ferrienterobactin receptor, were significantly upregulated in meropenem treatment group (Fig. 3C). Cluster analysis of related virulence proteins between the two groups showed that more virulence factors were enriched in the OMVs produced by meropenem-treated cultures (Fig. 3D). Cluster analysis was performed on the related stress proteins between the two groups, GroEL, and groL2 proteins were significantly upregulated in the meropenem-treated group (Fig. 3E).

**Fig 4:**
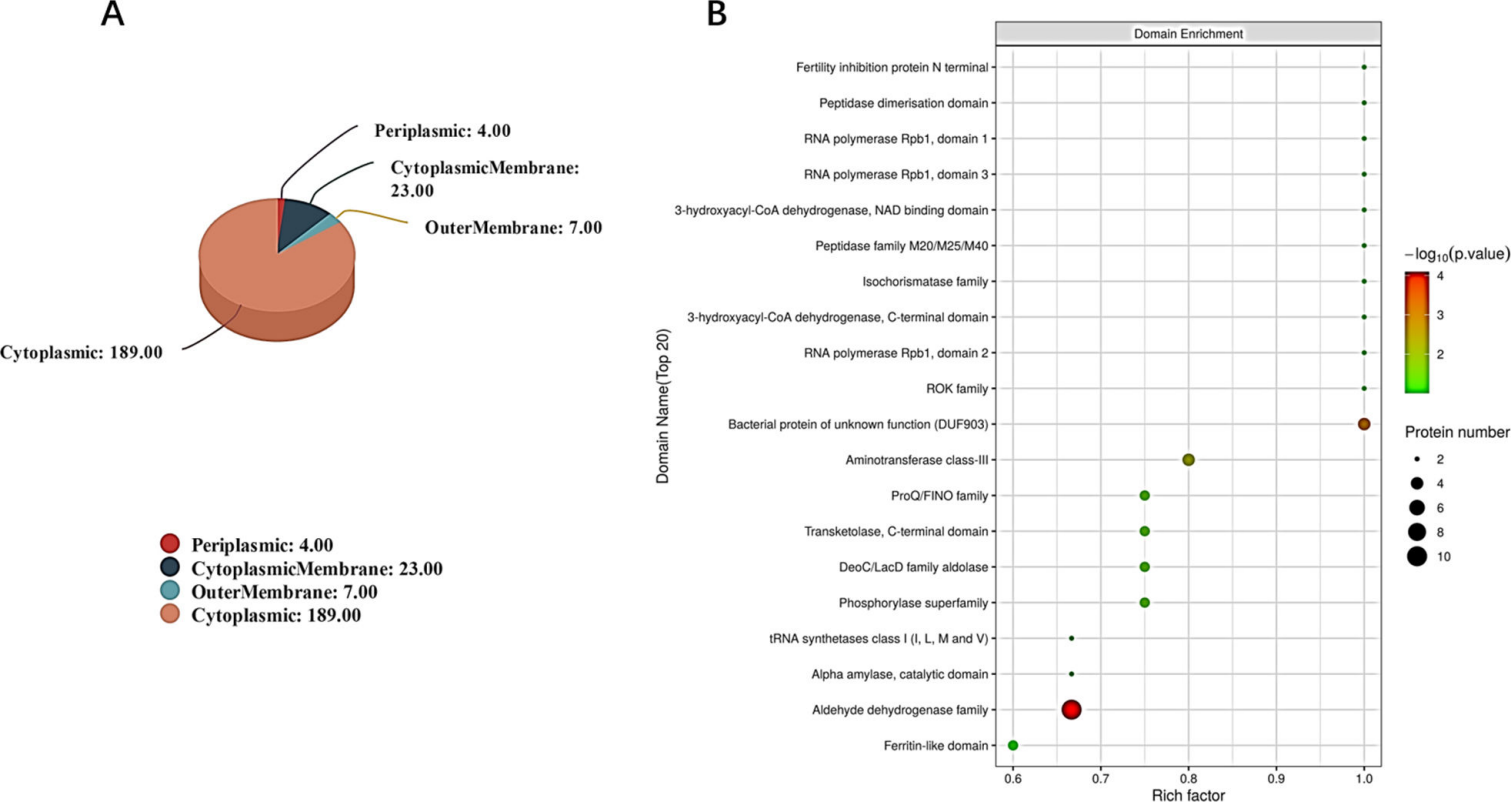
Pie chart of differential expression protein subcellular localization and structure domain enrichment analysis bubble diagram. (A) Use the subcellular structure prediction software CELLO to conduct subcellular localization analysis of all differentially expressed proteins. (B) Structure domain enrichment analysis bubble diagram. The abscissa in the figure was the enrichment factor (rich factor ≤ 1). The ordinate represents the statistical results of different proteins under each domain classification; The bubble color represents the significance of the enriched domain classification, the *P* value is calculated based on Fisher's exact test. The color gradient represents the size of the *P* value (−log10). The closer the color was to red, the smaller the *P* value was, and the higher the significance level of the enrichment under the corresponding domain classification.

The subcellular localization of OMVs was identiﬁed using the subcellular structure prediction software CELLO (19). For all differentially expressed proteins, outer membrane protein functionality was characterized as 189 cytoplasmic proteins, 23 cytoplasmic membrane proteins, seven outer membrane proteins, and four periplasmic proteins (Fig. 4A). Protein domains are two or more spatially distinct local regions in larger protein molecule due to the close association of adjacent super secondary structures on the polypeptide chain. Generally speaking, the interaction between protein and protein (or other small molecules) is often based on domain. The change of amino acid or modification in the domain may cause the change of key function of the protein, so the subsequent amino acid mutation function experiment can be used as a reference. The structural domain was predicted by interproscan software. The acetaldehyde dehydrogenase family has the highest significance level of enrichment under domain classification (Fig. 4B). According to previous research reports, deficiency of aldH genes in *K. pneumoniae* accelerates 1,3-propanediol production (20). Alcohol dehydrogenase mediated the production of acetaldehyde by *Helicobacter pylori,* which is likely the underlying mechanism of gastric injury (21).

**Fig 5:**
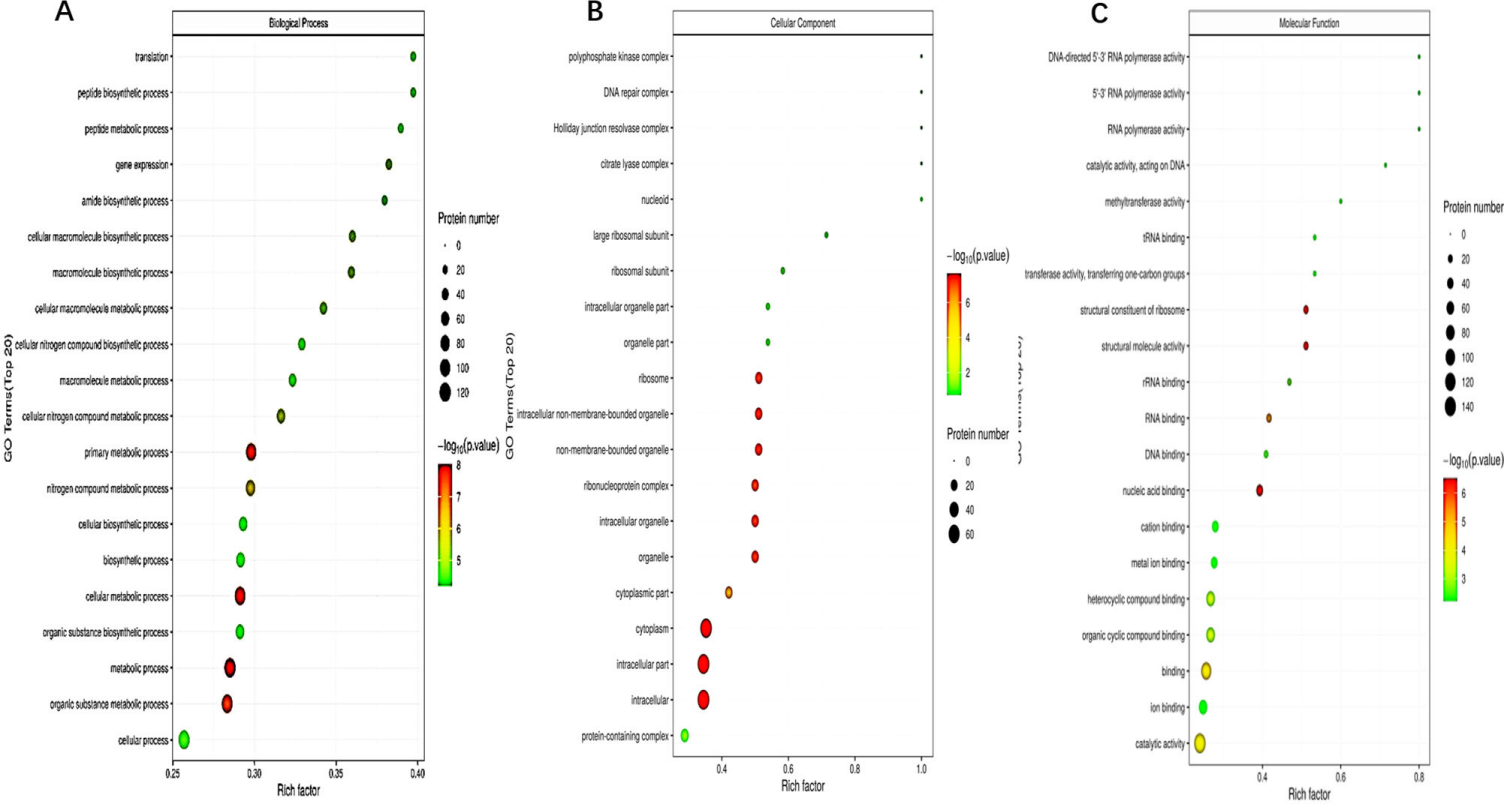
(A) Bubble diagram of GO function enrichment under biological process classification (Biological Process, BP), (B) Bubble diagram of GO function enrichment under cellular component classification (Cellular Component, CC), (C) Bubble diagram of GO function enrichment under molecular function classification (Molecular Function, MF). The abscissa in the figure was the enrichment factor (rich factor ≤ 1). The enrichment factor represents the proportion of the number of differentially expressed proteins annotated to a GO functional category to the number of all identified proteins annotated to the GO functional category. The ordinate represents the statistical results of different proteins under each GO functional classification; the bubble color represents the significance of the enriched GO function classification, and the *P* value was calculated based on Fisher's exact test. The color gradient represents the size of the *P* value (−log10). The closer the color was to red, the smaller the *P* value was, and the higher the significance level of the corresponding GO function classification enrichment. In general, the smaller the *P* value in the GO enrichment result (*P* < 0.05), the more statistically significant the enrichment of corresponding GO functional classification, and the number of differentially expressed proteins related to GO functional classification to some extent reflects the degree of influence of biological treatment on each classification in the experimental design.

Among the identiﬁed proteins, the majority of the differentially expressed proteins were involved in metabolic processes; cellular processes; as well as catalytic activity, and binding. Use Fisher’s exact test to conduct GO function enrichment analysis on differentially expressed proteins, revealing the overall function enrichment characteristics of all differentially expressed proteins. The highest enrichment significance of the differentially expressed proteins was associated with metabolic process, intracellular, cytoplasm, and nucleic acid binding (Fig. 5A through C). The speciﬁc proteins were chromosome partition protein MukB, 50S ribosomal protein L20, and methionine—tRNA ligase. OMVs may be related to host pathogenesis. This difference was expected considering the strain’s high sensitivity to antibiotic stress and environmental changes, as shown in previous studies (22).

**Fig 6:**
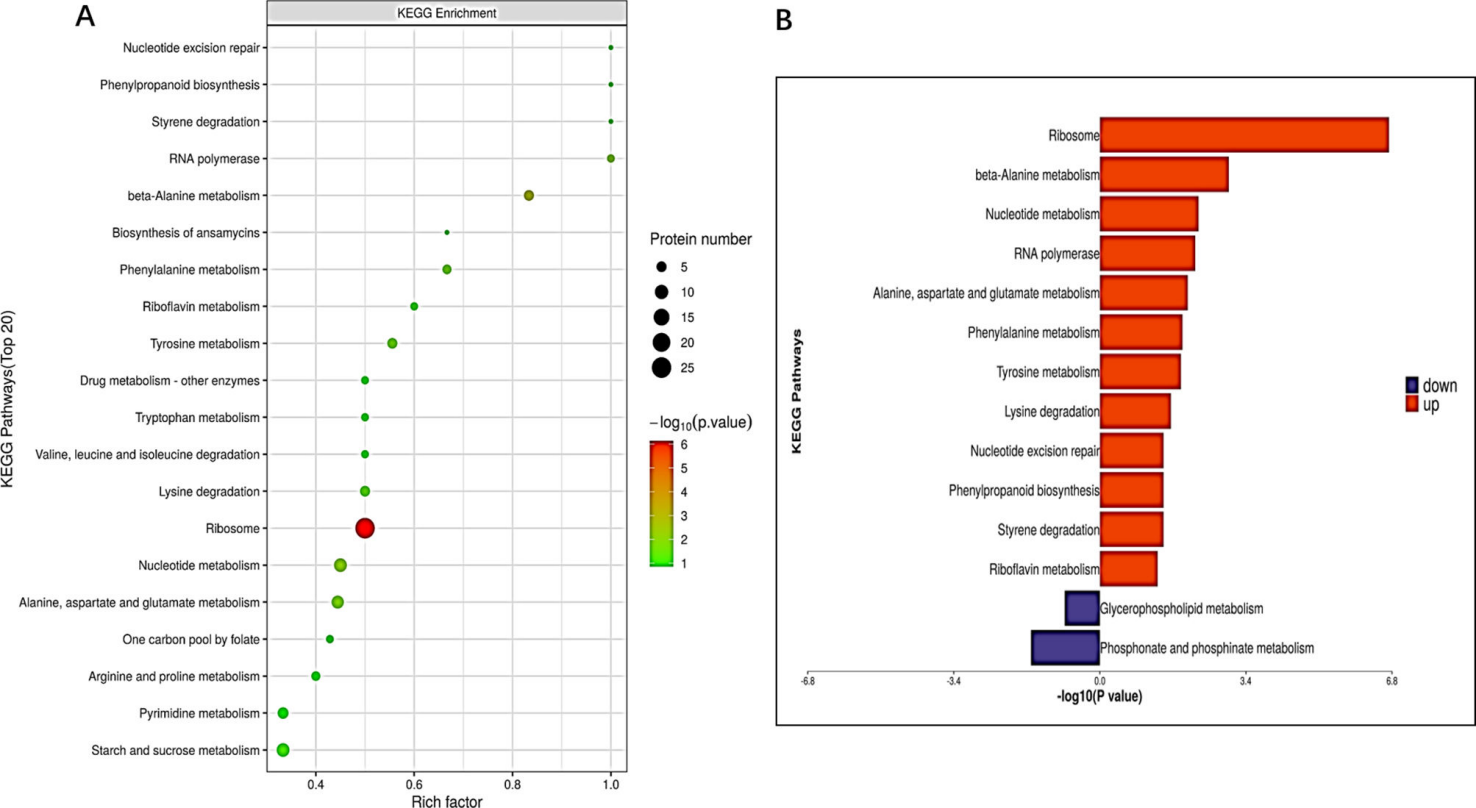
KEGG pathway enrichment bubble diagram and ribosome pathway mechanism diagram and butterfly diagram. (A) The abscissa in the figure was the enrichment factor (rch factor ≤ 1). The enrichment factor represents the proportion of the number of differentially expressed proteins annotated to the KEGG pathway category to the number of all identified proteins annotated to the category. The ordinate represents the statistical results of different proteins under each KEGG pathway; the bubble color represents the significance of the enriched KEGG pathway, and the *P* value was calculated based on Fisher's exact test. The color gradient represents the size of the *P* value (−log10). The closer the color was to red, the smaller the *P* value was, and the higher the significance level of the corresponding metabolic pathway enrichment was. (B) Pathway enrichment butterfly diagram of upregulation and downregulation differential proteins. The abscissa is the *P* value of Fisher's exact test (taking the logarithm with the base of 10), and the ordinate represents the path name. The pathways involved in upregulated and downregulated proteins are represented by red (right) and blue (left) bars.

In our study, we annotate protein analysis through the KEGG pathway database (Kyoto Encyclopedia of Genes and Genomes) (23). From the perspective of coordination of a series of proteins, this paper expounds on the change rules, such as changes in metabolic pathways, and more systematically and comprehensively analyzes biological processes, the pathogenesis of diseases, and the mechanism of drug action. Among the KEGG pathway annotation of the top 20 differentially expressed proteins, the ribosomal pathway had the largest number of differentially expressed proteins (Fig. 6A). The pathways involved in upregulated proteins are more concentrated in the synthesis and metabolism-related pathways of DNA or RNA, such as the ribosome, beta-alanine metabolism, nucleotide metabolism, RNA polymerase, alanine, aspartate and glutamate metabolism, phenylalanine metabolism, tyrosine metabolism, lysine degradation, nucleotide excision repair, phenylpropanoid biosynthesis, styrene degradation, and riboflavin metabolism (Fig. 6B).

**Fig 7:**
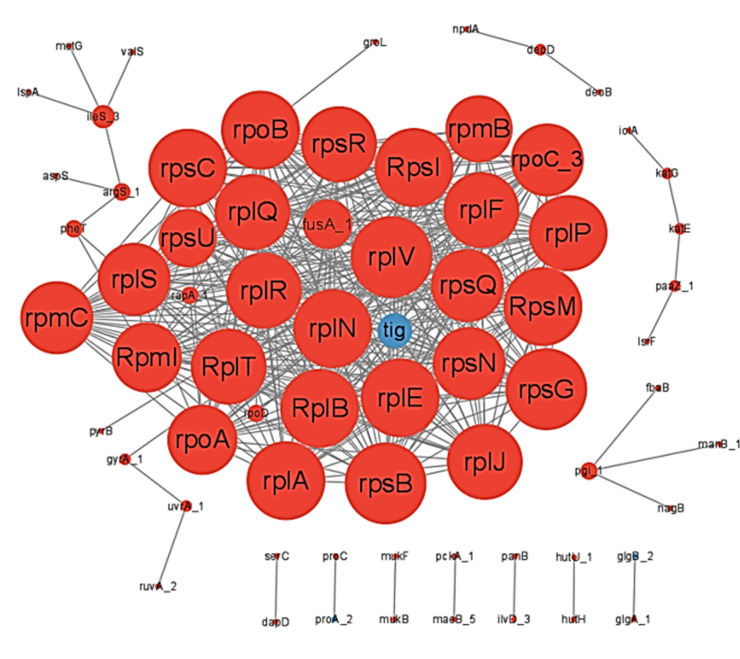
Interaction network diagram of differentially expressed proteins. Note: The circle node in the figure represents the differentially expressed protein, and the line represents the interaction between protein and protein. The color of the circle indicates the difference in protein expression (blue for downregulation and red for upregulation), and the size of the circle indicates the connectivity of the protein (i.e., the number of proteins directly interacting with a protein). Generally speaking, the greater the connectivity, the greater the disturbance to the whole system when the protein changes. It was more likely to be the key to maintaining the balance and stability of the system.

In the present study, we constructed a protein-protein interaction (PPI) network analysis of significantly differentially expressed proteins using a String database and conducted the analysis using Cytoscape software. One of the important pathways for proteins to function is through interactions with other proteins, and the use of protein-mediated pathways or the formation of complexes. The PPI results showed that significantly enriched proteins mainly include: ribosomal proteins such as rplV, rpsG, rpsM, and so on; virulence proteins such as mukB,manB, PGI, and so on; stress proteins such as grol (Fig. 7).

### Target proteins verified by a parallel reaction monitoring assay

Fourteen target proteins in the control or treatment groups were chosen for the parallel reaction monitoring (PRM) assay and subsequently successfully verified. These proteins were mainly related to virulence, such as bacterioferritin, rpoD, and PPA. The fold change comparison of the abundance of target proteins using PRM and label-free analysis is presented in Table 3, which indicates similar abundance change tendencies of the target proteins between the two analytical methods.

**TABLE 3:** The fold change of the abundance of the target proteins in the CRKP in the treatment and control groups using PRM and label-free analysis

Protein	Protein name	Gene name	Ratio value M-OMV/OMV	TTEST M-OMV/OMV
A0A080SLH1	4-hydroxy phenylacetate 3-monooxygenase	hpaB	0.4938	0.1586
A0A0C7KHW8	UvrABC system protein A	uvrA	0.6346	0.6175
A0A0E1C7D2	Bacterioferritin	KPNJ1_00416	8.6953	0.0291
A0A0E1CCW0	RNA polymerase sigma factor RpoD	rpoD	12.8164	0.0088
A0A0E1CL24	Glucose-6-phosphate isomerase	pgi	2.4569	0.2666
A0A0E1CNA2	Gamma-glutamyl phosphate reductase	proA	1.3903	0.6660
A0A2W0JDN4	Inorganic pyrophosphatase	ppa	7.2619	0.0064
A0A377ZP67	RNA-binding protein Hfq	hfq	5.5215	0.3178
A0A378E968	Xaa-Pro dipeptidase	pepQ	7.4951	0.0172
A6TGN6	50S ribosomal protein L1	rplA	10.4108	0.0046
B1Q1F2	16S rRNA (guanine(1405)-N (7))-methyltransferase	rmtB	0.4504	0.2444
Q9AGA6	6-phospho-alpha-glucosidase	aglB	6.8688	0.0177
R4YEM8	Uronate isomerase	uxaC	1.6955	0.2971
W8URL2	Aspartate carbamoyltransferase	pyrB	9.8066	0.0066

### Preliminary function verification experiment

**Fig 8:**
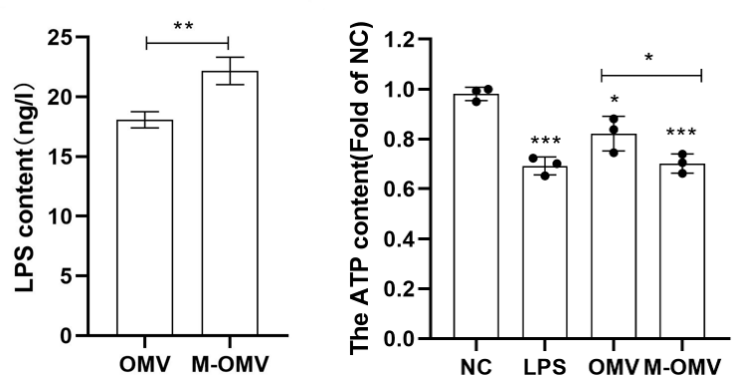
(Left) LPS content in both groups. Data correspond to the results from three independent experiments and were provided as means ± SD. (Right) The ATP content in RAW 264.7 cells. NC represents the negative control; LPS represents the positive control, the concentration of LPS was 1 µg/mL. OMV represents the OMV-treated group, and M-OMV represents the OMV-treated group induced by 4 µg/mL meropenem. There is a significant difference in ATP content between the OMV experimental group and the M-OMV experimental group, and the difference is statistically significant. More than three groups were analyzed by One-way ANOVA with Tukey’s multiple comparisons test and two groups were analyzed unpaired parametric t test. ****p < 0.0001, *** p < 0.001, ** p < 0.005, * p < 0.05, ns: not significant, p > 0.05.

We designed an *in vitro* experiment to investigate the effect of antibiotic-induced OMVs on host cell ATP concentration. Normally, when a cell is in a certain toxic state, ATP levels decrease, affecting the cell’s function. According to the proteomics results, the expression of virulence protein in the OMVs increased significantly after antibiotic induction. In our study, LPS was used as the positive control group (LPS concentration was 1 μg/mL), and the mean lipopolysaccharides (LPS) concentration (average of the three experiments) of the OMV and M-OMV were 18.09 ng/L and 21.35 ng/L, respectively (Fig. 8, left). The protein concentration of both OMV and M-OMV groups used in the experiment was 20 µg/mL. Our results showed that the LPS group, as a positive control group, had a significant difference from the negative control group in ATP content, the OMV group without antibiotic induction had a significant difference from the negative control group in ATP content, the M-OMV experimental group had a significant difference from the negative control group in ATP content, and there is a significant difference in ATP content between the OMV experimental group and the M-OMV experimental group, and the difference is statistically significant (Fig. 8, right).

## DISCUSSION

*K. pneumoniae* is one of the common pathogens of community-acquired and hospital-acquired infections (24). CRKP is a new pathogen that has emerged in hospitals, and it has been reported that the mortality rate of infected patients caused by CRKP is very high (25, 26). From *K. pneumoniae* to CRKP, there has been an important shift in the resistance of *K. pneumoniae*, with its emergence and spread greatly accelerated over the past few decades due to inappropriate and overuse of antibiotics (11). In this study, *K. pneumoniae* strains were isolated from clinical patients. Traditional antimicrobial characterization was carried out to disclose the resistant phenotype of the strain. The carbapenemase production test was positive, and the drug sensitivity test showed that it was resistant to meropenem and imipenem. Multilocus sequence typing (MLST) confirmed that the CRKP isolated strain in this study belongs to ST11, which produces KPC-2. Antibiotic treatment inhibits cell wall biosynthesis and induces membrane capsule formation, blistering cell death, and burst cell lysis, all of which have been observed in Gram-negative cells (27). However, few studies have discussed the molecular mechanisms of host disease caused by antibiotic abuse. We observed significant differentially expressed proteins in the OMV of CRKP after antibiotic induction, which are mainly involved in metabolic processes, toxicity, chaperones, resistance processes, and so on.

Most bacteria release OMVs containing specific molecules such as nucleic acids and proteins, which have a variety of functions (27). Over recent years, numerous lipidomic, proteomic, and RNA sequencing studies have revealed that vesicles composition varies depending on derived cell type, metabolic state, and disease status (28). Proteomic methods were used to study the changes in protein components in OMV after meropenem-induced CRKP. We found that meropenem-treated CRKP OMVs were more cytotoxic to cultured host cells. Proteins associated with virulence include PGI, manB, iron regulatory membrane protein, iutA, macB, TolC, lpt, and OPPA. According to previous studies, OPPA, an oligopeptide permease, has been shown to play multiple important roles in nutrition and toxicity among several bacteria (29). Bacterial OMVs play an important role as potent bacterial virulence factors and also play a role in cell survival (30, 31). Some studies have shown that OMVs can bind to certain antimicrobial molecules, leading to their inactivation or significantly promoting antibiotic resistance within biofilms (32, 33).

Among all the proteins identified in our study, the resistance-associated proteins were penicillin-binding protein 1B, EmrA, Na (+)/anti-drug transporter, arnT, arnA, arnC, blaKPC, blaSHV, SHV-112, macB, outer membrane channel protein, triple multidrug resistance system outer membrane protein, ompK36. Studies have shown that defects in *K. pneumoniae* pore proteins OmpK 35 and OmpK 36 may result in reduced susceptibility to carbapenems (34, 35). Bioinformatics studies such as KEGG enrichment and PPI network analysis suggest that they are involved in CAMP resistance and TCS. Furthermore, the metabolic status of bacteria plays a crucial role in mediating cell responses to antibiotic therapy (36). Recent studies have found that the metabolism of carbohydrates, starch, sucrose, arginine, and their derivatives proline plays an important role in the formation and maintenance of the biofilm (37–39). In this study, through the GO and KEGG pathway analysis, mass significantly differentially expressed proteins were involved in metabolic processes, including carbohydrate metabolism, amino acid metabolism, transcription, nucleotide, and lipid metabolism.

Importantly, in our study, among the proteins that were significantly differentially expressed after antibiotic induction. The protein with the highest significant difference is the Phenylacetic acid degradation bifunctional protein PaaZ. Relevant research showed that Paaz is located in the cytoplasm and participates in the phenylalanine metabolism pathway, belonging to the aldehyde dehydrogenase group A domain. Many bacteria use the enzyme PaaZ to degrade environmental pollutants. PaaZ is a bifunctional enzyme that catalyzes the ring opening of oxepin-CoA and converts it to 3-oxo-5,6-dehydrosuberyl-CoA (40). Among the significantly differentially expressed proteins, numerous proteins associated with carbohydrate metabolism, such as aglB, glgB, pckA, aceF, pykA, fbaB, deoB, and fbaB, highlight their underlying roles in *K. pneumoniae*. A former study reveals that mutants of the haloarchaeon that interfere with the biosynthesis of type IV pili, as well as aglB deletion mutants, lack obvious defects in biofilms formed in liquid cultures (41). In addition, chaperone proteins were also significantly differentially expressed under antibiotic stress and were involved in protein folding (42, 43). Proteins identified in our study include GroL-Gros chaperone complex, GroEL, chaperones DnaK, HSP 70, HtpG, and several peptidyl-prolyl isomerases, including periplasmic SurA chaperone. Expression of the chaperone protein GroEL helps prevent protein misfolding under stress conditions. As previously demonstrated, inappropriate antibiotic use against MDR bacteria enhances OMV secretion and upregulates the membrane presentation of GroEL, which promotes macrophage uptake of OMV, pyrosis, and release of pro-inflammatory cytokines and mediators (44).

Although OMV secretion requires massive energy expenditure, the benefits in life-and-death situations must be sufficient to allow OMV secretion to evolve. Analysis of proteins significantly differentially expressed in our study identified several fimbrial, outer membrane usher proteins fimC, and fimC belongs to adhesins and is a family of genes encoding virulence factors (45). RpoD is involved in flagella assembly.

OMV production can be used to allow the bacteria to survive long enough to establish a more permanent resistance (31). After the abuse of antibiotics, the OMVs of carbapenem-resistant *K. pneumoniae* express more survival factors. Llls and causes the cPS export system protein LptA may form a bridge between the inner membrane and the outer membrane via interactions with LptC and LptD, facilitating LPS translocation (https://www.uniprot.org/), which is essential to their survival. New questions have also been raised regarding the functional significance of LptA and LptC. Strictly regulated interactions between these connected subcomplexes may prove to be a valuable target for new antibiotic therapies for Gram-negative pathogens (46). In addition, the outer membrane channel, porin superfamily protein, and ferrienterobactin receptor are located in the outer membrane, located in TonB-dependent domains, these proteins are significantly differentially expressed in our proteomic results. Previous studies have shown that the downregulation of siderophore transporters, including tonB-dependent receptors, is one of the determinants of reduced *K. pneumoniae* biofilm formation (47).

Under certain special environmental conditions, such as the accumulation of protein aggregates or exposure to surface-damaging agents, OMVs have some ability to quickly clear misfolding membrane proteins or eliminate toxic substances (48). We have observed that many enzymes that are significantly differentially expressed in meropenem-induced OMVs are involved in the pathogenesis of infection, such as metallocarboxypeptidases, PPK, rmtB, AsnRS, budC, Hfq, PGI, manB, ompA, bacterioferritin, ferritin, and so on. The biological activity of metallocarboxypeptidases is regulated by inhibitors targeting metal-containing active sites. Some metal carboxypeptidase inhibitors are induced under stress conditions (49). The polyphosphate kinase (PPK) is the major enzyme in charge of inorganic polyphosphate (polyP) synthesis from ATP in many bacteria. Previous studies have shown that PPK or polyP plays a critical and important role in bacterial pathogenesis (50). In *K. pneumoniae*, the rare coexistence of plasmids encoding rmtB, armA, blaKPC-2, and iuc virulence operons poses a significant threat to clinical therapy (51). Asparaginyl-tRNA Synthetase (AsnRS) in *Leishmania major* and *Trypanosoma brucei* has been proven to show a significant role in survival and pathogenesis (52). Inactivation of the *Dickeya dadantii* bud gene indicated that bud mutants had significantly reduced toxicity (53). Hfq is an RNA-binding protein that promotes the interaction between sRNAs and their mRNA targets and is vital for the virulence of *K. pneumoniae* (54). Furthermore, PGI, manB, and ompA are virulence-associated proteins according to previous literature studies (55). OMVs also have specific virulence-related activity. For example, iron-related genes include bacterial ferritin and ferritin. The primary function of ferritins inside cells is to store iron. The previous studies found that *Escherichia coli* induces iron retention in A549 cells and causes the corresponding induction of the protein ferritin. This regulation of iron homeostasis may be mediated by oxidative stress (56, 57).

Similarly, in our study, OMPs were significantly differentially expressed after meropenem induction and previous studies have demonstrated that OMPs are also important for molecular transport and pathogenesis (58). The OmpA pore protein interacts with peptidoglycans and is therefore responsible for the stability of the bacterial outer membrane. Previous research on *A. baumannii* has shown that the OmpA protein in OMVs can trigger intense proinflammatory responses (59, 60). More than that, OmpC overexpression exacerbates bacterial resistance, especially to β-lactamines (61).

As the most important energy molecule, ATP plays a crucial role in the physiological and pathological processes of cells. Changes in ATP levels can affect cell function. Mitochondrial ATP production is normally reduced by sublethal concentrations of different pharmacological compounds (62). According to the proteomic results, we examined, the expression of extracellular vesicular toxic proteins was significantly increased after meropenem induction, while OMVs induced a significant decrease in host cell ATP concentrations. Therefore, we demonstrate that meropenem produces stress proteins that increase the pathogenicity of host cells.

In conclusion, proteomics analysis indicated that meropenem-induced OMVs of *K. pneumoniae* showed enrichment of proteins/enzymes associated with virulence factors, pathogenicity, drug resistance, stress, bacterial survival, and cellular metabolism. The PPI network of proteins identified from meropenem-induced and control OMVs was constructed using the String database and analyzed using Cytoscape software. Our study showed that the growth of CRKP induced by meropenem MIC conditions was significantly correlated with PPI network interactions. Therefore, these significantly differentially expressed proteins are of great significance for exploring effective control strategies against infections of CRKP. Our research hints that inappropriate use of antibiotics can lead to a decrease in host cell ATP and an increase in cell pathogenicity. The mechanism may be that antibiotics enhance the virulence protein secretion of *K. pneumoniae* OMVs, resulting in host disease. These results will shed light on the pathogenicity of *K. pneumoniae* and help prevent and control *K. pneumoniae* infections. Therefore, elucidating the biological functions of OMV protein components is of great significance for elaborating the pathogenic mechanism of OMVs, and developing diagnostic tools, vaccines, and effective anti-pathogenic antibiotics. The future direction of OMVs proteomic research on Gram-negative bacteria under the condition of overuse of antibiotics is expected to promote the development of basic science and clinical science.

## MATERIALS AND METHODS

### Bacterial strain, growth conditions, and antimicrobial treatment

The *K. pneumoniae* strain used in our study was a clinically isolated CRKP (63). The isolated individual colonies were inoculated into lysogeny broth (LB) (Solarbio, China) with or without meropenem (4 µg/mL) at 37°C, with gentle shaking at 180 rpm until an optical density at 600 nm (OD_600_) of 0.8 is achieved (Every 200 mL of bacterial solution requires approximately 8 h of growth.).

### Whole-genome sequencing

Genomic DNA was extracted from *K. pneumoniae* clinical isolates using a bacterial genomic DNA extraction kit (Tiangen, China), and sequenced by the PacBio Technologies platform. The strain had a 5,398,959 bp chromosome and five main plasmids with sizes of 132,440 base pairs (bp), 173,750 (bp), 69,379 (bp), 10,060 (bp), and 5,596 (bp), respectively.

### Transmission electron microscopy detection of bacteria

In the ultra-clean platform, 200 µL bacterial solution was added to 200 mL of LB culture medium and was shaken the bacteria overnight at 37°C. After cultivation, the bacteria were centrifuged for 5 min and sediment was collected. The sample was blown and fixed with Dan electron microscope fixing solution, and fixed overnight in a 4°C refrigerator. The sample was pre-embedded in agar. The bacterial sample was embedded, sliced, and detected by transmission electron microscopy, and the formation of a single OMV in the bacteria was observed.

### Antimicrobial susceptibility test

The antimicrobial susceptibility of the *K. pneumoniae* strain was determined using the broth microdilution method according to the Clinical and Laboratory Standards Institute (CLSI) guidelines (64). Briefly, the MICs of antibiotics were determined using the Vitek 2 system and ASTGN card (bioM é rieux, France) for the isolated CRKP strains. The Kirby Bauer disk diffusion method was used as a supplementary drug sensitivity test. Strains were designated susceptible (S), intermediate (I), and resistant (R) based on their respective MIC values and CLSI-deﬁned interpretive criteria.

### Isolation and puriﬁcation of OMVs

The bacterial OMVs were isolated from culture supernatants according to previous studies with some modiﬁcation (65). In short, the bacterial culture supernatants were collected and filtered through a 0.22-µm sterile filter. Then filtrates were concentrated by ultrafiltration through a 100,000 NMWC (Merck Millipore, Germany) column at 4°C. Concentrated supernatants were further ultracentrifuged at 150,000 × *g* for 120 min at 4°C using a 70 Ti rotor (Beckman Coulter, USA). The obtained vesicular pellets were collected as crude OMVs and were resuspended in 500 µL of phosphate-buffered saline (PBS). Then, the crude OMVs were purified by OptiPrep (60% iodixanol; Axis-Shield, Norway) density gradient ultracentrifugation (66). About 12 mL of OptiPrep density gradient solutions with 3 mL 30%, 3 mL 20%, 3 mL 10%, and 3 mL 5% density gradients was added from bottom to top, respectively. About 500 µL of crude OMVs was deposited on top of the density gradient of a 13.2 mL ultracentrifuge tube and centrifuged at 100,000 × *g* for 18 h at 4°C with a SW40 Ti rotor. The centrifuged liquid was fractionated from top to bottom in a tube of every 5 mL and centrifuged at 100,000 × *g* for 1 hour at 4°C. Then, the OMVs layer was recovered, resuspended by PBS, and centrifuged again at 100,000 × *g* for 2.5 h at 4°C. Then, the purified OMVs layer was resuspended in PBS. The OMVs suspension was then filtered again through a 0.22-µM membrane filter and cultured on LB agar plates to ensure sterility.

### Determination of proteins and lipopolysaccharides in OMVs

OMVs proteins were quantified by microscale BCA Test Kit (63). According to the manufacturer’s instructions, use the LPS ELISA kit (mlbio, China) to detect the presence of LPS in OMVs samples (63).

### Transmission electron microscopy

The morphology of OMVs was observed and imaged by transmission electron microscopy (TEM) (Hitachi, Japan)(63). In brief, 10 µL of samples was placed on copper mesh for 5–10 min, and excess liquid was absorbed by the ﬁlter paper. The vesicle suspension was stained with 2% uranyl acetate (10 µL) and visualized by using the HT7700 transmission electron microscope (Hitachi, Japan).

### Nanoparticle tracking analysis

Particle count and size distribution of OMVs were measured by NTA using a ZetaView_Particle Metrix (Particle Metrix, PMX-120, Germany). NTA software was used to measure the concentration of nanoparticles (particles/mL).

### Sodium dodecyl sulfate polyacrylamide gel electrophoresis (SDS-PAGE)

About 1 µg of protein for each sample was mixed with 5× loading buffer and boiled for 5 min. The proteins were separated on 10% SDS-PAGE gel (constant current 80 V, 30 min; constant current 120 V, 45 min). Protein bands were visualized by silver staining of the Biorad imager.

### OMVs protein proﬁle by tandem mass spectrometry (MS/MS)

LC-MS/MS analysis was performed on a timsTOF Pro mass spectrometer (Bruker) (63).

### Proteomics bioinformatic analysis and software used

Cluster 3.0 (http://bonsai.hgc.jp/~mdehoon/software/cluster/software.htm) and Java Treeview software (http://jtreeview.sourceforge.net) were used to perform hierarchical clustering analysis. A heat map was often presented as a visual aid in addition to the dendrogram. The localization of OMVs was identiﬁed using the subcellular structure prediction software CELLO. Use the domain prediction software Interprescan-5.25–64.0 (67) to predict the domain of differentially expressed proteins. Protein sequences were searched using InterProScan software to identify protein domain signatures from the InterPro member database Pfam. Draw GO annotation results through the R language program. After the annotation step, the studied proteins were analyzed against the online KEGG database (http://geneontology.org/) to retrieve their KEGG orthology identification and subsequent mapping to pathways in KEGG (63). Enrichment analysis was applied based on Fisher’s exact test, considering the whole quantified proteins as the background data set. Benjamini-Hochberg correction for multiple testing was further applied to adjust derived *P* values. Only functional categories and pathways with *P* values under a threshold of 0.05 were considered significant. The false discovery rate of proteomic analysis is 0.01.

### Protein-protein interaction network analysis

The PPI information was retrieved from the IntAct molecular interaction database (68) (http://www.ebi.ac.uk/intact/) by gene symbols or STRING software (http://string-db.org/). The results were downloaded in the XGMML format and imported into Cytoscape 3.9.1 software to visualize and further analyze functional protein-protein interaction networks. Furthermore, the degree of each protein was calculated to evaluate the importance of the protein in the PPI network.

### Parallel reaction monitoring analysis

Select proteins that are significantly more abundant and closely related to virulence for PRM validation. Perform LC-PRM/MS (Q-Empirical HF and Easy nLC 1200 system, Thermo Fisher Scientific) assays to verify the levels of selected target proteins obtained through unlabeled analysis.

Use Skyline v3.5.0 (69) to analyze the PRM raw data of processing and control samples. Quantify the signal intensity of each unique peptide or target protein for each sample and standardize it as a standard reference (70).

### Cell culture

Mouse mononuclear cell line RAW 264.7 was cultured in Dulbecco Modified Eagle Medium (DMEM) supplemented with 10% heat-inactivated FBS, 100 U/mL penicillin, and 100 µg/mL streptomycin at 37°C in a humidified incubator with 5% CO_2_.

### ATP concentration determination

Raw264.7 cells were seeded into six-well plates (4 × 10^5^ cells per well) in full growth media and incubated overnight for 24 h before corresponding. Then, OMV/M-OMV containing 20 µg/mL protein was added and incubated at 37°C for 24 h. Intracellular ATP levels were measured using a commercially available intracellular ATP assay kit (Beyotime, China), according to the manufacturer’s instructions. Briefly, the collected cells were lysed and centrifuged at 12,000 × *g* for 5 min at 4°C. After that, an aliquot of the supernatant was taken and reacted with ATP detection solution in a 96-well plate (Biosharp, China). Luminescence was detected using a PerkinElmer MultiMode plate Reader. The ATP level was presented as nanomoles per milligram of protein.

### Statistical analysis

All experiments were repeated three times. GraphPad Prism 8.0(GraphPad Software, USA) was used to perform the statistical analyses, and the standard deviations (SDs) were calcu­lated. A P value < 0.05 was used to determine Statistical significance. The data in the text were represented as mean±SD, and more than three groups were analyzed by One-way ANOVA with Tukey’s multiple comparisons test and two groups were analyzed unpaired parametric t test. ****p < 0.0001, *** p < 0.001, ** p < 0.005, * p < 0.05, ns: not significant, p > 0.05.

## Data Availability

The mass spectrometry proteomics data have been deposited to the ProteomeXchange Consortium (http://proteomecentral.proteomexchange.org) via the iProX partner repository (71, 72) with the data set identifier PXD047553.

## AFTER EPUB

*[This article was published on 18 January 2024 with errors in Fig. 8. The figure and its legend were corrected in the current version, posted on 24 January 2024.]*
